# Worksite health promotion and social inequalities in health

**DOI:** 10.1016/j.ssmph.2020.100543

**Published:** 2020-01-17

**Authors:** Anne C. van der Put, Jornt J. Mandemakers, John B.F. de Wit, Tanja van der Lippe

**Affiliations:** a Department of Sociology, Utrecht University, Padualaan 14, 3584 CH, Utrecht, the Netherlands; b Interdisciplinary Social Science: Public Health, Utrecht University, Padualaan 14, 3584 CH, Utrecht, the Netherlands

**Keywords:** Europe, Worksite health promotion, Health inequalities, Education, Self-rated health, Multilevel structural equation modelling, Mediation, Moderation

## Abstract

It is well-documented that higher educated employees have better health than the lower educated. The workplace has been put forward as a contributor to this inequality. We extend previous work on workplace characteristics that could influence employee health by asking to what extent workplace health promotion (WHP) can account for the relation between education and health. Two ways in which WHP may relate to health inequalities are addressed: higher educated employees may be more likely to use WHP than lower educated employees and the effect of WHP on health may be stronger for higher educated than for lower educated employees. Using data from the European Sustainable Workforce Survey which contains information on over 11000 employees in 259 organisations, we test whether three types of WHP mediate or moderate the relation between education and health: healthy menus, sports facilities and health checks. We find that higher educated employees are in better health and that use of WHP positively relates to health. Use of healthy menus and sports facilities in the workplace can contribute to increasing health inequalities, as lower educated employees are less likely to make use of these. Health checks could contribute to diminishing health inequalities, as lower educated employees are more likely to use them compared to higher educated employees. The effect of WHP is not contingent on education. We advise stimulating lower educated employees to make more use of WHP, which can contribute to decreasing health inequalities.

## Highlights

• Healthy workplace menus enlarge health inequalities among employees.
• Use of sports facilities at work is not related to health inequalities.
• Health checks do not mitigate inequalities, despite more use by the lower educated.
• The effect of WHP is not contingent on education, and all employees benefit equally.
• Lower educated employees should be stimulated to use WHP more.

## 1 Introduction

It has been well-documented that higher educated people have better health than those who are lower educated (Thrane, 2006; von dem Knesebeck, Verde, & Dragano, 2006; Vonneilich, Lüdecke, & von dem Knesebeck, 2019). There are three main explanations for why this is the case: lower educated people are said to live in less favourable material conditions, engage in less healthy behaviours and find themselves in less favourable psychosocial environments compared to the higher educated (Mackenbach et al., 2015). Within the workplace, these explanations come together. Previous research has shown that among many other health-aversive working conditions, lower educated employees are more often exposed to toxic chemicals, more often engage in heavy lifting, and have less autonomy than higher educated employees, which contributes to worse health (Dieker et al., 2019; Hämmig, Gutzwiller, & Kawachi, 2014; Meneton et al., 2018).

Although aspects of the organisations in which employees work may also have an effect on health, these have received less attention in the literature (Marklund, Bolin, & von Essen, 2008). One such aspect is Worksite Health Promotion (WHP), interventions targeting health and healthy behaviours among employees. Workplaces are considered promising places for health promotion as adults spend a majority of their waking hours at work, and WHP has been widely adopted to improve public health, in particular in the post-industrial societies of the global North (Jørgensen, Villadsen, Burr, Mortensen, & Holtermann, 2015). There is no systematic overview of the extent to which WHP is offered in Europe, but previous studies found that about 30–40% of European organisations provide healthy menus in the workplace cafeteria, 30–50% promote physical activity, and 65–75% offer health checks (Van der Put & Mandemakers, 2019; Verra, Benzerga, Jiao, & Ruggeri, 2019). These are among the most prevalent types of WHP (Goetzel, Shechter, Ozminkowski, Tabrizi, & Roemer, 2007), and the focus of this paper.

Previous research has assessed whether WHP affects the health of *all* employees, and shown that healthy menus, sports facilities and health checks at work can have beneficial but modest effects on employee health (e.g. Conn, Hafdahl, Cooper, Brown, & Lusk, 2009; Maes et al., 2012; Rongen, Robroek, van Lenthe, & Burdorf, 2013). However, it is not yet known how WHP relates to health inequalities (Bull, Gillette, Glasgow, & Estabrooks, 2003). Firstly, WHP could potentially increase health inequalities if higher educated employees are more likely to use it and as a result have better health. Secondly, research on health promotion interventions shows that higher educated people may benefit more from such interventions than lower educated people (Adams, Mytton, White, & Monsivais, 2016). This may also apply to WHP if it affects the health of higher educated employees more strongly than that of lower educated employees. This paper therefore asks whether WHP accounts for the relation between education and health and whether WHP is more effective for higher than lower educated employees.

Our study contributes to previous research in a several ways. Firstly, many work factors have been studied in relation to health inequalities. We extend the current literature by looking at WHP. While some other work factors that are related to health, such as work demands and autonomy, may be inherently linked to specific jobs, this is not the case for WHP, which could potentially be used by all employees regardless of their level of education. Interventions that are available to all are more effective in diminishing health interventions than interventions targeted at specific subgroups of employees, such as smokers (Adams et al., 2016). Given that healthy menus, sports facilities and health checks can be used by all employees, they may be an effective way of mitigating health inequalities compared to job characteristics previously studied.

Secondly, one of the reasons why it is unknown whether WHP contributes to the relation between education and health is because most studies on WHP rely on data from only one or a few organisations in one sector and so cannot incorporate educational differences in workforce compositions (Bull et al., 2003). Socio-demographic characteristics are seldom addressed, and studies that do mostly include higher educated employees (Anderson et al., 2009). An exception is the work by Sorensen et al. (2005), but their sample was too small to detect differences. Some research focused specifically on WHP targeted at lower educated employees (e.g. Lassen, Bruselius-Jensen, Sommer, Thorsen, & Trolle, 2007), but this cannot provide insight in whether lower educated employees use and benefit more from WHP than higher educated employees. We use unique cross-sectional multilevel data from the European Sustainable Workforce Survey (Van der Lippe et al., 2016) which contains over 11,000 employees nested in 259 organisations in nine European countries. This allows us to examine variation in WHP among organisations, while addressing the role of socio-demographic characteristics, notably education. We believe our study makes a valuable contribution to clarifying the role of WHP in health inequalities among employees.

Thirdly, our study has clear social relevance for employers. Health inequalities affect organisations in terms of the health of their workforce, absenteeism rates and productivity (Eurofound, 2012). We study policies that are actually implemented in organisations rather than test interventions newly designed by researchers; they thus better reflect reality (Bull et al., 2003). When we know how WHP relates to health inequalities, this can inform action on how to tackle health differences. For example, should employers encourage lower educated employees to make use of WHP that is available to all or target WHP specifically towards lower educated employees? By shedding light on how WHP relates to health inequalities we hope that our results can inform policy makers and employers on effective ways to reduce those.

## 2 Background

**Fig. 1:**
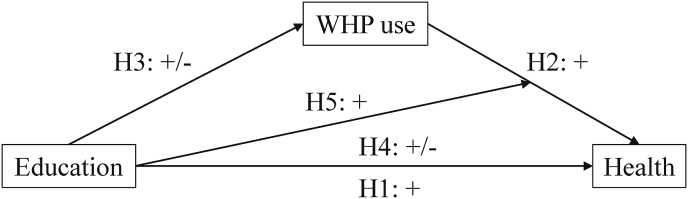
The conceptual model showing the direct effects between education and health (H1), WHP use and health (H2), education and WHP use (H3) as well as the expected mediation (H4) and moderation (H5).

Given the well-documented relation between education and health (see for example Dieker et al., 2019; Hämmig et al., 2014; Thrane, 2006), we expect that higher educated employees have better health than lower educated employees (H1). The main aim of our study is to examine whether WHP can explain (part of) this relation. There are two ways in which WHP may relate to health inequalities: (1) higher educated employees may be more likely to use WHP than lower educated employees, and (2) the effect of WHP on health may be stronger for higher educated than lower educated employees. We explain these pathways in more detail after elaborating why WHP may increase health. A schematic overview of our expected hypotheses can be seen in Fig. 1.

### 2.1 Worksite health promotion and health

There are several ways in which WHP can contribute to employee health. Firstly, WHP may make employees more aware of their health, so they pay more attention to it (Hendriksen, Snoijer, De Kok, Van Vilsteren, & Hofstetter, 2016). If employees eat healthily at work, they may also extend this behaviour to their private lives. Secondly, WHP can increase knowledge of the advantages of a healthy lifestyle, resulting in more employees making healthy choices (Anderson et al., 2009). Thirdly, by offering more opportunities for healthy behaviour, WHP can contribute to decreasing the cost of healthy choices (Engbers, Van Poppel, Chin A Paw, & Van Mechelen, 2005). For example, financial contributions by employers towards a gym membership will make being physically active less costly for employees. Fourthly, environmental cues, such as visibly placing salads in the workplace cafeteria, can influence unconscious behaviour and support the development of more healthy habits (Kahn-Marshall & Gallant, 2012). Previous studies have shown that WHP can have beneficial but modest effects on employee health. Employees who make use of WHP have been found to have healthier diets (Maes et al., 2012), be more physically active (Conn et al., 2009), reduce weight (Anderson et al., 2009), and have better health in general (Rongen et al., 2013). We thus expect that use of WHP contributes to better health (H2).

### 2.2 Education and access to WHP

Educational health inequalities between employees may be partly attributable to differential access to WHP. On the one hand, organisations may be more likely to make WHP available to lower educated employees as they are in higher need of such organisational policies given their generally higher work-related health risks and overall worse health (Bagwell & Bush, 1999). Providing WHP to employees who have much or more to gain from it is likely beneficial for organisations in terms of productivity and absenteeism (Goetzel & Ozminkowski, 2008). On the other hand, higher educated employees may work more often in larger organisations, which have more resources for WHP implementation (Goetzel & Ozminkowski, 2008; Stiehl et al., 2018). Additionally, WHP may be more often targeted towards higher educated employees because these are seen as more valuable for the organisation (Hammerback et al., 2015). With the exception of Emmons et al. (2000), who found that organisations with a larger share of lower educated employees more often offer smoking cessation programs, most studies suggest lower educated employees have less access to WHP (Brack, 2008; Harris, Huang, Hannon, & Williams, 2011; Parrish et al., 2018).

### 2.3 Education and use of WHP

Even when lower educated employees have access to WHP, there are several reasons why they may be less likely to use it. Firstly, they usually have less human capital, which can make them less successful in dealing with information, and less familiar with benefits of eating healthily and being physically active (Burton, Turrell, & Oldenburg, 2003). Lower educated employees are not less likely to know that WHP exists in their organisation (Van der Put & Mandemakers, 2019) but they may be unaware that using WHP can help them become healthier (Rongen et al., 2014, Rongen et al., 2014). In addition, they may not attribute illness to personal health behaviour and not think they need WHP (Burton et al., 2003).

Secondly, lower educated employees may have less opportunity at work to use WHP. For instance, bringing one's own lunch may be cheaper than buying a healthy lunch in the worksite cafeteria (Raulio, Roos, & Prättälä, 2012), and lower educated employees with fewer financial resources may refrain from using healthy menus. Engaging in physical activity at work, or making use of a sponsored subscription, requires time and effort, while these barriers are likely especially relevant for the lower educated (Bukman et al., 2014). In addition, the working conditions of lower educated employees may also hinder them in using WHP. Lower educated employees tend to be overrepresented in jobs with little autonomy, while this enables WHP use (Jørgensen, Villadsen, Burr, Punnett, & Holtermann, 2016). They are also more likely to work in shifts or away from the organisation, such as fire fighters, which also hampers WHP use (Ranby et al., 2011).

Thirdly, social norms arising from colleagues, peers and family members indicate what (healthy) behaviour is appropriate, and if favourable may induce employees to use WHP (Smith & Christakis, 2008). Lower educated employees are more likely to find themselves in an unhealthy social environment, both at work and outside, and more often come into contact with unhealthy behaviours (Bukman et al., 2014; Pampel, Krueger, & Denney, 2010). This may make them less likely to engage in healthy behaviours and to use WHP. Higher educated employees, in contrast, more often find themselves in social situations in which healthy behaviour is the norm. This may, however, apply less to health checks, as lower educated employees may be more likely to work in sectors where having one's health checked occasionally is the norm (Walters, Wadsworth, & Quinlan, 2013), if not required.

Earlier research has shown that lower educated employees are less likely to make use of a variety of WHP (Dobbins, Simpson, Oldenburg, Owen, & Harris, 1998; Kilpatrick, Blizzard, Sanderson, Teale, & Venn, 2015; Raulio, Roos, Mukala, & Prättälä, 2007; Robroek, van Lenthe, van Empelen, & Burdorf, 2009) We hence expect that lower educated employees are less likely to use healthy menus (H3a) and sports facilities (H3b). This is not necessarily the case for health checks at work, which may be easier to do when offered at work and in some cases may be compulsory for professions and in sectors in which mainly lower educated employees work (Walters et al., 2013). We hence expect lower educated employees to be more likely to use health checks (H3c). In view of the expected differential WHP use by employees of different educational levels, we furthermore hypothesise that the use of healthy menus (H4a) and sports facilities (H4b) will contribute to increased health inequalities, while health checks (H4c) contribute to diminishing them.

### 2.4 Education and effect of WHP

The second way in which WHP may relate to health inequalities is if the effect of using WHP is different for higher educated than for lower educated employees. Previous studies have shown that the health of both lower (Lassen et al., 2007) and higher educated (Gretebeck, Bailey, & Gretebeck, 2017) employees can benefit from WHP, but it is unknown whether benefits differ according to educational level.

Research on health promotion shows that interventions that target whole populations rather than specific individuals, and rely on people engaging with information and voluntary behaviour change, are more likely to benefit the higher educated (Adams et al., 2016). This may also be the case for WHP. Notably, healthy menus, sports facilities and health checks in the workplace are examples of such population interventions, as they are available to all in a particular setting. However, employees need to know about these interventions and their benefits, as well as use them consistently.

Furthermore, self-interest utility theory poses that interventions are likely to be successful when employees find them personally useful and have experienced the benefits (Casper & Harris, 2008). Higher educated employees may find WHP more useful (van Lenthe, Jansen, & Kamphuis, 2015), and be more open to interventions that support behaviour change (Backholer et al., 2014). On the other hand, WHP may be more relevant for lower educated employees because of their generally worse health (Bagwell & Bush, 1999). For example Sorensen et al. (2005) found that lower educated employees experienced bigger improvements in healthy eating and physical activity after participating in WHP than higher educated employees, who were already behaving more healthily. However, WHP may be better tailored to the needs of higher educated employees because of the health behaviours they focus on (Rongen et al., 2014, Rongen et al., 2014). Supporting this possibility, Rongen et al. (2013) report that higher educated employees benefit more from WHP. We hence expect that the effect of using WHP to be larger for higher educated employees (H5).

## 3 Data and methods

### 3.1 Sample

We used cross-sectional data from the European Sustainable Workforce Survey (ESWS), undertaken in 2015/2016 in nine European countries: Bulgaria, Finland, Germany, Hungary, the Netherlands, Portugal, Spain, Sweden and the United Kingdom (Van der Lippe et al., 2016). The ESWS is a multilevel survey which includes reports from employees, department managers and HR managers. Organisations were selected using stratified random sampling by country, sector (manufacturing, healthcare, higher education, transport, telecom and financial services) and size (up to 100 employees, 101–249 employees and more than 250 employees). When an organisation did not want to participate, a similar organisation based on these characteristics was approached. Employees and managers were contacted at work to complete the self-report questionnaire. The study has been declared to be in line with all ethical requirements. In participating organisations, response rates were 61% for employees, 81% for department managers and 98% for HR managers. In total, 11,011 employees in 259 organisations participated in the survey.

We excluded employees for which we had no response from the HR manager, given that we lacked information on organisational characteristics (N = 301 employees in 8 organisations). We used listwise deletion of respondents with missing data on any of the included variables (N = 647, mainly missing on self-rated health). Our total sample consisted of 10063 employees in 251 organisations. As availability differs by WHP type, sample sizes differ between the analyses related to different types of WHP.

### 3.2 Variables

Employees were asked to self-report their perceived *health* on a scale ranging from 1 (very good) to 5 (very bad). Although self-rated health may not give a complete view of someone's health, this measure has been found to be a good predictor of morbidity and mortality in Europe (Dieker et al., 2019; Hämmig et al., 2014). Scores were reversed so that higher scores indicated better health.

To measure level of *education*, we used years of education. Education is the key to one's position in the social stratification system and precedes occupational status and income, two other often-used indicators of socioeconomic status (von dem Knesebeck et al., 2006). Employees were asked for their highest completed level of education, based on the International Standard Classification of Education. Levels of education per country were matched to formal years of education (OECD, 2012).

*WHP use* was measured by employee self-reports. Employees first had to indicate whether they thought the three types of WHP were available in their organisation: catering or cafeteria menus offering healthy nutrition, sport facilities at work or a financial contribution towards a sports activity outside the workplace, and health checks to assess employees’ current state of health. Only when employees reported a policy to be available, they could indicate whether they used it (yes = 1, no = 0). When employees reported a policy to be unavailable or did not know of its existence, they were considered as not using it. We created three variables, one for each type of WHP.

### 3.3 Statistical analyses

To examine the relationship between education, health and WHP, we controlled our analyses for *gender* (female = 1) and a curvilinear effect of *age* as these both have been found to be related to self-rated health (Marmot, Allen, Bell, Bloomer, & Goldblatt, 2012). Age was divided by 10 for ease of interpretation. There may be differences in WHP availability between countries (Van der Put & Mandemakers, 2019) which could impact the extent to which employees can make use of WHP. We therefore controlled for *WHP availability* as reported by the HR manager and *country*. Descriptive statistics of all variables are shown in Table 1.

**Table 1:** Descriptive statistics.

Variables	M	SD	Range
Self-rated health	3.88	0.74	1–5
Education	13.65	3.14	3–21
Healthy menus use	0.29		0–1
Sports facilities use	0.17		0–1
Health checks use	0.35		0–1
Age	42.14	11.03	14–77
Female	0.56		0–1
Healthy menus availability	0.45		0–1
Sports facilities availability	0.53		0–1
Health checks availability	0.65		0–1
Country			
United Kingdom	0.07		0–1
Germany	0.09		0–1
Finland	0.07		0–1
Sweden	0.10		0–1
The Netherlands	0.22		0–1
Portugal	0.11		0–1
Spain	0.07		0–1
Hungary	0.12		0–1
Bulgaria	0.14		0–1
N employees	10063		
N organisations	251		

Because employees who work in organisations may share certain attributes, we applied a multilevel structure to allow for this nesting of the data (Hox, 2010). Specifically, we fitted multilevel generalised structural equation models (Preacher, Zyphur, & Zhang, 2010). We first fitted empty two-level models for use of each type of WHP and health as outcomes. These models show how much variation can be explained by differences between organisations. We then fitted mediation models, one for each type of WHP, including the control variables. We disentangled the direct effect (education on health) from the indirect effect (education on health through WHP use), and tested if the indirect effect can explain part of the relation between education and health. Indirect effects were calculated using the product-of-coefficients approach and consist of a multiplication of the effect of education on WHP use and of WHP use on health. Total effects are the sum of direct and indirect effects. We used logistic regression equations for the analyses examining the relation between education and WHP use, given that WHP use is dichotomous, and used linear regression equations for the other analyses. In addition to assessing WHP as a mediator, we also examined whether the effect of WHP on health is different for lower and higher educated employees accounting for possible differences in WHP use. We therefore added interaction terms between education and WHP use to estimate conditional indirect effects (Preacher, Rucker, & Hayes, 2007).

## 4 Results

**Fig. 2:**
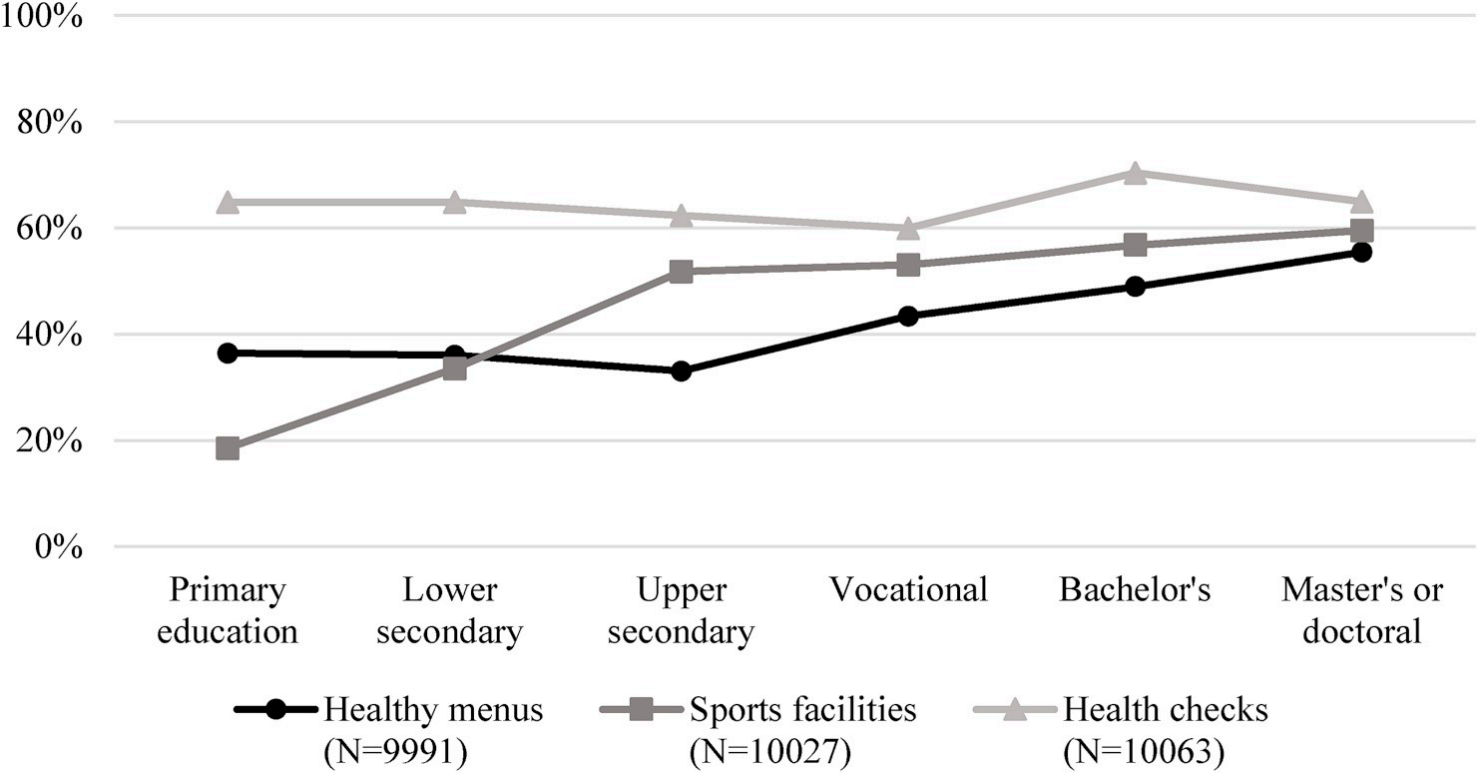
Availability for healthy menus, sports facilities and health checks by highest completed level of education.

On average, 45% of employees had healthy menus available in their workplace, 53% had access to sports facilities and 65% had the possibility to have a health check. As Fig. 2 shows, higher educated employees tended to have more access to healthy menus and sports facilities, but not to health checks. Regarding WHP use, we found that healthy menus are used by 29% of employees, sports facilities by 17%, and health checks by 35%. Empty models showed that 54%, 67% and 63% of the variation in use of healthy menus, sports facilities and health checks, respectively, is explained by differences between organisations. The variation between organisations for self-rated health is 4%.

**Fig. 3:**
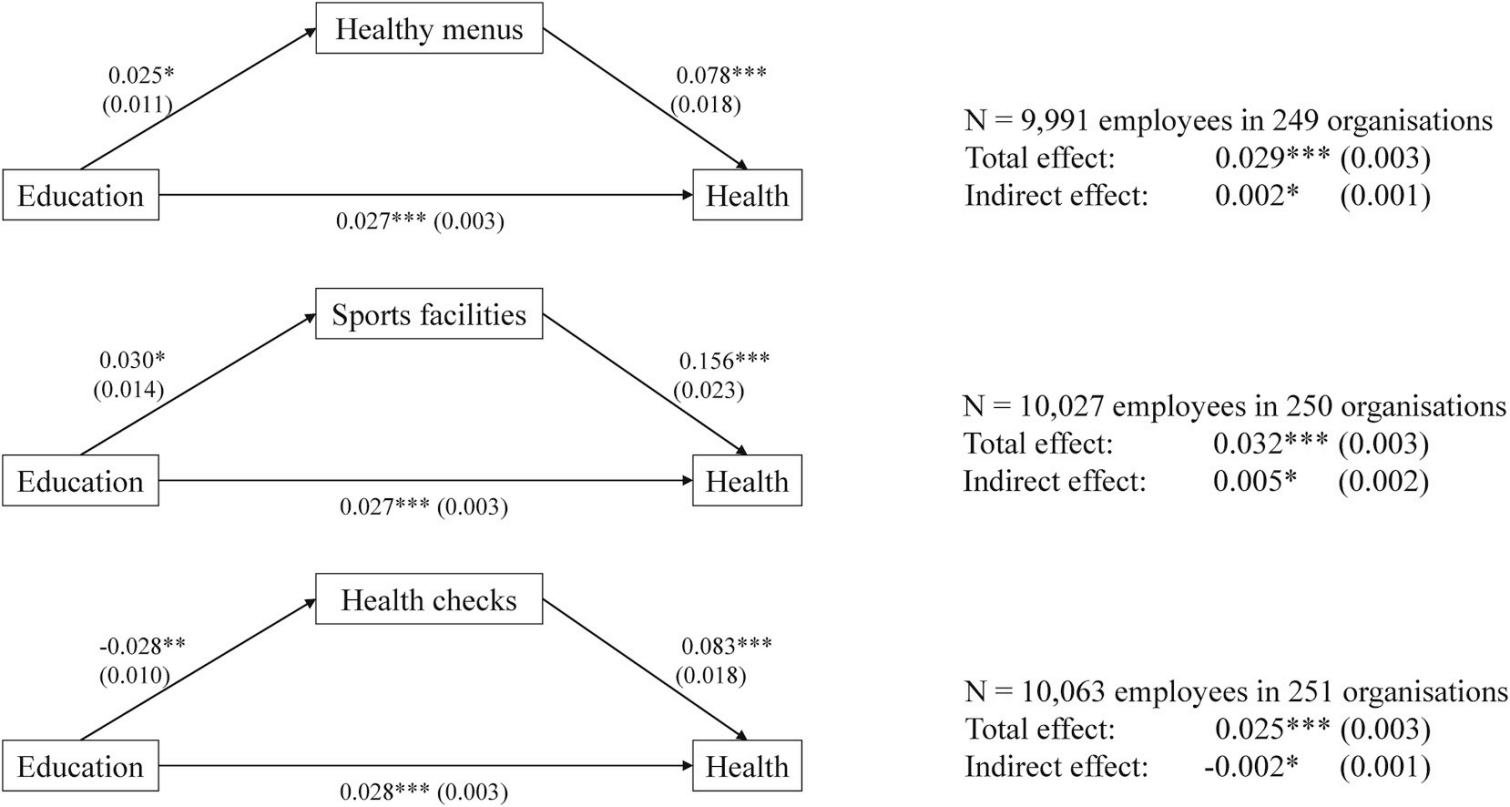
Structural equation models with mediation of WHP in the relation between education and health. Results for control variables (gender, age, age^2^, size, sector and country) can be found in Table S.1 and Table S.2. Standard errors are shown in parentheses. Total and indirect effects of WHP are summarised with standard errors for each model. *p < .05, **p < .01, ***p < .001.

Fig. 3 shows the results of the analyses of the relations between education, WHP and health. In support of our first hypothesis, in all models we find that higher educated employees rate their health as better than lower educated employees. For every additional year of education, employees score about 0.03 point higher on the 5-point self-rated health scale. Results also show that for each type of WHP, employees who use WHP rate their health as better than employees who do not use WHP. In support of hypothesis 2, employees who use healthy menus, sports facilities or health checks on average rate their health 0.08, 0.16 and 0.08 points higher, respectively.

Fig. 3 shows that our expectations that compared to higher educated employees, lower educated employees are less likely to make use of healthy menus (H3a) and sports facilities (H3b), but more likely to use health checks (H3c), are supported.

Fig. 3 also presents the total effect of education on health, broken down into the direct effect and indirect effect, that is, through WHP use. We find support for our fourth hypothesis: use of healthy menus, sports facilities and health checks are significant partial mediators of the association between education and health. As higher educated employees are more likely to use healthy menus and sports facilities, this contributes to increasing health inequalities, while lower educated employees are more likely to use health checks, which contributes to diminishing health inequalities. These mediation effects are however small: healthy menus explain 1.4% of education-related inequalities, sports facilities 1.2% and health checks 0.6%.

**Fig. 4:**
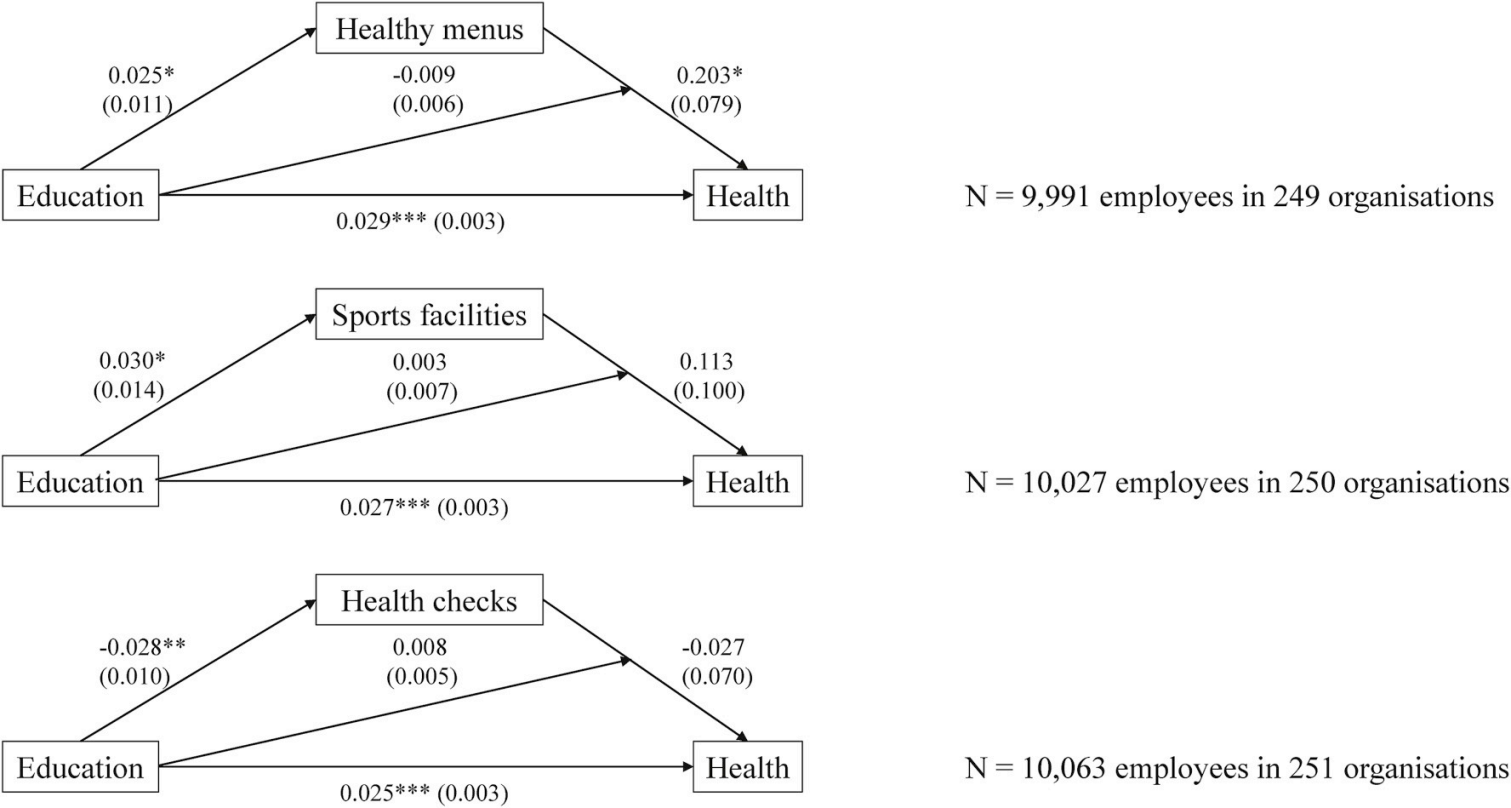
Structural equation models with moderated mediation of WHP in the relation between education and health. Results for control variables (gender, age, age^2^, size, sector and country) can be found in Table S.1 and Table S.2. Standard errors are shown in parentheses. *p < .05, **p < .01, ***p < .001.

Furthermore, we expected that the effect of WHP on health would be larger for higher-educated employees. The results of the analyses including the interaction between education and WHP use are shown in Fig. 4. These indicate that the hypothesised moderation effects are not significant; the effect of WHP on health is not contingent on education and findings do not support hypothesis 5.

### 4.1 Additional analyses

In addition to education, occupational status and income also reflect an individual's position in the social hierarchy (Dieker et al., 2019), so we ran our analyses using these variables instead of education to assess the robustness of findings as a reflection of social status-related health inequalities. Results remained the same when using occupation as an indication of social status. We found there is no relation between WHP used and income, and hence no possible mediation.

There are many organisational characteristics that could be related to both WHP use and health (Jørgensen et al., 2016), and we therefore also re-ran our analyses while controlling for flexibile working arrangements, financial situation, competitive work culture, size and organisational sector. Results remained the same for healthy menus and health checks. However, the relation between education and use of sports facilities became marginally significant (p = 0.066), as did the mediation (p = 0.076).

Employees who use one type of WHP may be more likely to also use other types of WHP, so we also re-ran our analyses for the number of WHP used (0–3). We found no relation between education and number of WHP used, underscoring the importance of examining each type of WHP separately. Results for these additional analyses can be found in the supplementary material.

## 5 Discussion

The aim of this study was to assess to what extent WHP can account for the relation between education and health, and whether WHP may be more effective for higher than lower educated employees. Health inequalities among employees have been well-documented (Dieker et al., 2019). Many different work factors have been studied as explanations for why higher educated employees may have better health than lower educated employees, and we extend this by studying WHP. WHP has been widely adopted as a means to improve public health and can be used by all employees, regardless of their educational background (Adams et al., 2016; Jørgensen et al., 2015). While previous research has addressed whether WHP can affect the health of all employees, it has however not addressed differences in effects between higher and lower educated employees. Using data from over 10,000 employees in 251 organisations in nine countries, we assessed whether use of healthy menus, sports facilities and health checks mediates the relation between education and health, and if the association between WHP and health differs by level of education. Our findings provide insight into if and how WHP can diminish health inequalities.

We found that, as expected, higher educated employees rate their health as better than lower educated employees. This is in line with many prior studies (Dieker et al., 2019; Hämmig et al., 2014; Thrane, 2006; von dem Knesebeck et al., 2006; Vonneilich et al., 2019), and the main contribution of our study is its assessment of the role of WHP in this association. We also conclude that as WHP use relates to better health, this could potentially help in diminishing health inequalities. However, higher educated employees appear to have more access to WHP (especially healthy menus and sports facilities) than lower educated employees, and one way for to reduce education-related health inequalities would be to increase access of lower educated employees to WHP. In addition, higher and lower educated employees differ in the extent to which they use WHP when controlling for availability, and so only making WHP *available* to employees is not enough to reduce health inequalities.

Lower educated employees are less likely to make use of healthy menus, and because of this, healthy menus in the workplace can compound existing health inequalities. Raulio et al. (2012) suggested that lower educated employees may less often use healthy menus because these are more expensive, but our additional analyses showed that income is not related to use of healthy menus in the workplace. Alternatively, lower educated employees may have less opportunities to attend the worksite cafeteria because of shift work, not working at the organisation's main venue(s) (e.g. truck drivers) or a belief that because their jobs are more often physically demanding, they need more energy-rich but unhealthier food (Backman, Gonzaga, Sugerman, Francis, & Cook, 2011; Hulsegge et al., 2016; Passey et al., 2014). Providing lower educated employees with the possibility to visit a workplace cafeteria during work hours and stimulating them to choose healthy food options may help increase use of healthy menus and, through that, reduce health inequalities.

We find that the use of sports facilities also mediates the relation between education and health, as lower educated employees are less likely to use sports facilities. Reasons for this could be similar to why lower educated employees are less likely to use healthy menus, that is, fewer opportunities to use sports facilities during work hours or having a physically demanding job which may discourage them from additional physical activity. To reduce health inequalities, the use of sports facilities among lower educated employees may need to be promoted.

We found that lower educated employees are more likely to make use of health checks, which may reduce health inequalities. Part of the reason why use among them is higher may be that lower educated employees may work more often in sectors where such checks are obligatory (Walters et al., 2013). Additionally, compared to higher educated, lower educated employees may be more likely to have their health checked when they already experience health issues (Bukman et al., 2014). This would help lower educated employees in finding out they have health issues and help them address these to protect or promote their health.

We found the relationship between WHP and health is not moderated by education, meaning that WHP works equally well for lower and higher educated employees *when used*. Given that we found that lower educated employees are less likely to use healthy menus and sports facilities while these do contribute to better health, the main challenge to reducing health inequalities through WHP is to encourage lower educated employees to make use of available WHP. Merely offering WHP is likely insufficient to promote health and reduce health inequalities, as it relies on individual agency, and work on other types of health interventions has shown that this increases health inequalities (Adams et al., 2016). It is important for employers to actively stimulate and enable lower educated employees to make use of WHP. As lower educated employees are not less likely to know about the existence of WHP (Van der Put & Mandemakers, 2019), a main challenge may relate to providing these employees with the opportunities to fit WHP into their work schedule and to motivate them to make healthy choices. Creating healthy norms within an organisation can contribute to achieving this, for example by offering healthy snacks at events during office hours and installing sit/stand desks in offices.

We note that the effects of WHP we find are only small. Previous studies also found small health effects of WHP (e.g. Rongen et al., 2013). Additionally, there are many factors that contribute to education-related health inequalities, and the aim of our study was to examine whether WHP could be one of those. We therefore do not claim that if lower educated employees use WHP, health inequalities will disappear, but we do believe that WHP is part of the solution. In line with Rose's theorem, we posit that small effects for individuals can potentially have substantial relevance for public health (Adams et al., 2016).

We want to note several limitations to our study. Firstly, as our data are cross-sectional, we cannot assess potential reversed causality. Notably, organisations with more higher educated employees may be more likely to implement WHP because these employees are more likely to request WHP and are more actively engaged with organisational policy to provide WHP (Goetzel et al., 2007). Furthermore, higher educated employees tend to be healthier and healthier employees may be more likely to use WHP (e.g. Rongen et al., 2013). Randomised controlled trials have however shown that WHP use precedes health outcomes (Maes et al., 2012), but these studies did not address health inequalities. We regard our study as making an important contribution to understanding the role of employees’ socio-demographic characteristics in the potential health benefits of WHP, and recommend future studies further examine whether WHP affects health inequalities as our findings suggest.

Secondly, our measure of self-rated health may not may not optimally capture the diverse aspects of employees’ experienced health. Also, we did not include any objective health indicators nor did we assess health behaviours related to diet, physical activity and alcohol consumption. Health behaviours are proximal determinants of health, and relations between education and self-rated health may likely run through these behaviours (Toch et al., 2014). However, previous studies have shown that self-rated health is a good indicator of mortality and morbidity (Dieker et al., 2019) Future research should nevertheless assess whether WHP is also associated with social inequalities in health behaviours as well as objective indicators.

Lastly, our measures of WHP do not fully capture what WHP entails. For example, health checks may include a thorough examination of several health aspects or only consist of measuring blood pressure and BMI. In addition, we only know whether employees made use of WHP in the last 12 months but not how often, which implies that WHP use may reflect occasional or irregular use as well as frequent or regular use. While other studies have also employed this measure (e.g. Jørgensen et al., 2015), a more detailed assessment of what WHP entails and how it is used is recommended.

A strength of our study is that it is among the first to explicitly address how WHP might be related to health inequalities by studying the role of education in the use and effect of WHP. We made use of rich data allowing us to take into consideration that organisations differ in their workforce composition, which is an improvement to other studies that only focus on one or a few organisations in one sector (Bull et al., 2003). In addition, we studied three types of WHP rather than just one and find specific results per type of WHP, suggesting it is important to account for the variety in WHP on offer. Future research could assess how other types of WHP may relate to health inequalities. Furthermore, the inclusion of a large number of organisations enabled us to account for differences in availability of WHP. Some have argued that WHP may affect employees differently as not all employees have equal access to WHP (Parrish et al., 2018), but by controlling for differential availability, we found differences remain in WHP use by education level.

## 6 Conclusion

Education-related health inequalities are ubiquitous, and work-related differences are an important explanation for why higher educated employees may have better health than lower educated employees. We examined to what extent WHP can account for the association between education and health, and whether WHP may be more effective for higher than lower educated employees. This study is among the first to assess how employees’ socio-demographic characteristics affect the use of WHP. We conclude that the use of healthy menus and sports facilities in the workplace can contribute to increasing health inequalities, as lower educated employees are less likely to make use of these. Health checks could contribute to diminishing health inequalities, as lower educated employees are more likely to use them compared to higher educated employees. Importantly, we found that the association between WHP and health was similar for all employees. Given this general health-promoting potential of WHP, we recommend organisations and workplace health promoters to encourage lower educated employees to make use of WHP, to contribute to mitigating health inequalities.

## Funding source declaration

This work was supported by the European Research Council (ERC Grant Agreement n. 340045). This funding source has had no influence on the preparation of this article.

## CRediT authorship contribution statement

**Anne C. van der Put:** Conceptualization, Methodology, Formal analysis, Writing - original draft, Visualization. **Jornt J. Mandemakers:** Conceptualization, Writing - review & editing, Supervision. **John B.F. de Wit:** Conceptualization, Methodology, Validation, Writing - review & editing. **Tanja van der Lippe:** Conceptualization, Methodology, Validation, Writing - review & editing, Investigation, Data curation.

## Declaration of competing interest

None.

## References

[bib1] Adams J., Mytton O., White M., Monsivais P. (2016). Why are some population interventions for diet and obesity more equitable and effective than others? The role of individual agency. PLoS Medicine.

[bib2] Anderson L.M., Quinn T.A., Glanz K., Ramirez G., Kahwati L.C., Johnson D.B. (2009). The effectiveness of worksite nutrition and physical activity interventions for controlling employee overweight and obesity. A systematic review. American Journal of Preventive Medicine.

[bib3] Backholer K., Beauchamp A., Ball K., Turrell G., Martin J., Woods J. (2014). A framework for evaluating the impact of obesity prevention strategies on socioeconomic inequalities in weight. American Journal of Public Health.

[bib4] Backman D., Gonzaga G., Sugerman S., Francis D., Cook S. (2011). Effect of fresh fruit availability at worksites on the fruit and vegetable consumption of low-wage employees. Journal of Nutrition Education and Behavior.

[bib5] Bagwell M.M., Bush H.A. (1999). Health conception and health promotion in blue collar workers. AAOHN Journal.

[bib6] Brack A.B. (2008). Differences in employee multidimensional health by gender, age, and educational level. Journal of Workplace Behavioral Health.

[bib7] Bukman A.J., van Baak M.A., Meershoek A., Renes R.J., Feskens E.J.M., Teuscher D. (2014). Perceptions on healthy eating, physical activity and lifestyle advice: Opportunities for adapting lifestyle interventions to individuals with low socioeconomic status. BMC Public Health.

[bib8] Bull S.S., Gillette C., Glasgow R.E., Estabrooks P. (2003). Work site health promotion research: To what extent can we generalize the results and what is needed to translate research to practice?. Health Education & Behavior.

[bib9] Burton N.W., Turrell G., Oldenburg B. (2003). Participation in recreational physical activity: Why do socioeconomic groups differ?. Health Education & Behavior.

[bib10] Casper W.J., Harris C.M. (2008). Work-life benefits and organizational attachment: Self-interest utility and signaling theory models. Journal of Vocational Behavior.

[bib11] Conn V.S., Hafdahl A.R., Cooper P.S., Brown L.M., Lusk S.L. (2009). Meta-analysis of workplace physical activity interventions. American Journal of Preventive Medicine.

[bib12] Dieker A.C., Burdorf A., Hulsegge G., Proper K.I., Ket J.C., van der Beek A.J. (2019). The contribution of work and lifestyle factors to socioeconomic inequalities in self-rated health ‒ a systematic review. Scandinavian Journal of Work, Environment & Health.

[bib13] Dobbins T.A., Simpson J.M., Oldenburg B., Owen N., Harris D. (1998). Who comes to a workplace health risk assessment?. International Journal of Behavioral Medicine.

[bib14] Emmons K.M., Thompson B., McLerran D., Sorensen G., Linnan L., Basen-Engquist K. (2000). The relationship between organizational characteristics and the adoption of workplace smoking policies. Health Education & Behavior.

[bib15] Engbers L.H., Van Poppel M.N.M., Chin A Paw M.J.M., Van Mechelen W. (2005). Worksite health promotion programs with environmental changes: A systematic review. American Journal of Preventive Medicine.

[bib16] Eurofound (2012). Health and well-being at work: A report based on the fifth European working conditions survey. Dublin. https://www.eurofound.europa.eu/sites/default/files/ef_publication/field_ef_document/ef1302en.pdf.

[bib17] Goetzel R.Z., Ozminkowski R.J. (2008). The health and cost benefits of work site health-promotion programs. Annual Review of Public Health.

[bib18] Goetzel R.Z., Shechter D., Ozminkowski R.J., Tabrizi M.J., Roemer E.C. (2007). Promising practices in employer health and productivity management efforts: Findings from a benchmarking study. Journal of Occupational and Environmental Medicine.

[bib19] Gretebeck K.A., Bailey T., Gretebeck R.J. (2017). A minimal contact diet and physical activity intervention for white-collar workers. Workplace Health & Safety.

[bib20] Hammerback K., Hannon P.A., Harris J.R., Clegg-Thorp C., Kohn M., Parrish A. (2015). Perspectives on workplace health promotion among employees in low-wage industries. American Journal of Health Promotion.

[bib21] Hämmig O., Gutzwiller F., Kawachi I. (2014). The contribution of lifestyle and work factors to social inequalities in self-rated health among the employed population in Switzerland. Social Science & Medicine.

[bib22] Harris J.R., Huang Y., Hannon P.A., Williams B. (2011). Low–socioeconomic status workers: Their health risks and how to reach them. Journal of Occupational and Environmental Medicine.

[bib23] Hendriksen I.J.M., Snoijer M., De Kok B.P.H., Van Vilsteren J., Hofstetter H. (2016). Effectiveness of a multilevel workplace health promotion program on vitality, health, and work-related outcomes. Journal of Occupational and Environmental Medicine.

[bib24] Hox J.J. (2010). Multilevel analysis. Techniques and applications.

[bib25] Hulsegge G., Boer J.M.A., van der Beek A.J., Verschuren W.M.M., Sluijs I., Vermeulen R. (2016). Shift workers have a similar diet quality but higher energy intake than day workers. Scandinavian Journal of Work, Environment & Health.

[bib26] Jørgensen M.B., Villadsen E., Burr H., Mortensen O.S., Holtermann A. (2015). Does workplace health promotion in Denmark reach relevant target groups?. Health Promotion International.

[bib27] Jørgensen M.B., Villadsen E., Burr H., Punnett L., Holtermann A. (2016). Does employee participation in workplace health promotion depend on the working environment? A cross-sectional study of Danish workers. BMJ Open.

[bib28] Kahn-Marshall J.L., Gallant M.P. (2012). Making healthy behaviors the easy choice for employees. Health Education & Behavior.

[bib29] Kilpatrick M., Blizzard L., Sanderson K., Teale B., Venn A. (2015). Factors associated with availability of, and employee participation in, comprehensive workplace health promotion in a large and diverse Australian public sector setting. Journal of Occupational and Environmental Medicine.

[bib30] von dem Knesebeck O., Verde P.E., Dragano N. (2006). Education and health in 22 European countries. Social Science & Medicine.

[bib31] Lassen A., Bruselius-Jensen M., Sommer H.M., Thorsen A.V., Trolle E. (2007). Factors influencing participation rates and employees' attitudes toward promoting healthy eating at blue-collar worksites. Health Education Research.

[bib32] van Lenthe F.J., Jansen T., Kamphuis C.B.M. (2015). Understanding socio-economic inequalities in food choice behaviour: Can maslow's pyramid help?. British Journal of Nutrition.

[bib33] Mackenbach J.P., Kulhánová I., Bopp M., Deboosere P., Eikemo T.A., Hoffmann R. (2015). Variations in the relation between education and cause-specific mortality in 19 European populations: A test of the “fundamental causes” theory of social inequalities in health. Social Science & Medicine.

[bib34] Maes L., Van Cauwenberghe E., Van Lippevelde W., Spittaels H., De Pauw E., Oppert J.M. (2012). Effectiveness of workplace interventions in Europe promoting healthy eating: A systematic review. The European Journal of Public Health.

[bib35] Marklund S., Bolin M., von Essen J. (2008). Can individual health differences be explained by workplace characteristics?-A multilevel analysis. Social Science & Medicine.

[bib36] Marmot M., Allen J., Bell R., Bloomer E., Goldblatt P. (2012). WHO European review of social determinants of health and the health divide. The Lancet.

[bib37] Meneton P., Hoertel N., Wiernik E., Lemogne C., Ribet C., Bonenfant S. (2018). Work environment mediates a large part of social inequalities in the incidence of several common cardiovascular risk factors: Findings from the Gazel cohort. Social Science & Medicine.

[bib38] OECD (2012). PISA 2012 technical report program for international assessment.

[bib39] Pampel F.C., Krueger P.M., Denney J.T. (2010). Socioeconomic disparities in health behaviors. Annual Review of Sociology.

[bib40] Parrish A.T., Hammerback K., Hannon P.A., Mason C., Wilkie M.N., Harris J.R. (2018). Supporting the health of low socioeconomic status employees. Journal of Occupational and Environmental Medicine.

[bib41] Passey D.G., Robbins R., Hegmann K.T., Ott U., Thiese M., Garg A. (2014). Long haul truck drivers' views on the barriers and facilitators to healthy eating and physical activity: A qualitative study. International Journal of Workplace Health Management.

[bib42] Preacher K.J., Rucker D.D., Hayes A.F. (2007). Addressing moderated mediation hypotheses: Theory, methods, and prescriptions. Multivariate Behavioral Research.

[bib43] Preacher K.J., Zyphur M.J., Zhang Z. (2010). A general multilevel SEM framework for assessing multilevel mediation. Psychological Methods.

[bib44] Ranby K.W., MacKinnon D.P., Fairchild A.J., Elliot D.L., Kuehl K.S., Goldberg L. (2011). The PHLAME (promoting healthy lifestyles: Alternative models' effects) firefighter study: Testing mediating mechanisms. Journal of Occupational Health Psychology.

[bib45] Raulio S., Roos E., Mukala K., Prättälä R. (2007). Can working conditions explain differences in eating patterns during working hours?. Public Health Nutrition.

[bib46] Raulio S., Roos E., Prättälä R. (2012). Sociodemographic and work-related variation in employees' lunch eating patterns. International Journal of Workplace Health Management.

[bib47] Robroek S.J.W., van Lenthe F.J., van Empelen P., Burdorf A. (2009). Determinants of participation in worksite health promotion programmes: A systematic review. International Journal of Behavioral Nutrition and Physical Activity.

[bib48] Rongen A., Robroek S.J.W., Burdorf A. (2014). The importance of internal health beliefs for employees' participation in health promotion programs. Preventive Medicine.

[bib49] Rongen A., Robroek S.J.W., van Ginkel W., Lindeboom D., Pet M., Burdorf A. (2014). How needs and preferences of employees influence participation in health promotion programs: A six-month follow-up study. BMC Public Health.

[bib50] Rongen A., Robroek S.J., van Lenthe F.J., Burdorf A. (2013). Workplace health promotion: A meta-analysis of effectiveness. American Journal of Preventive Medicine.

[bib51] Smith K.P., Christakis N.A. (2008). Social networks and health. Annual Review of Sociology.

[bib52] Sorensen G., Barbeau E., Stoddard A.M., Hunt M.K., Kaphingst K., Wallace L. (2005). Promoting behavior change among working-class, multiethnic workers: Results of the healthy directions - small business study. American Journal of Public Health.

[bib53] Stiehl E., Shivaprakash N., Thatcher E., Ornelas I.J., Kneipp S., Baron S.L. (2018). Worksite health promotion for low-wage workers: A scoping literature review. American Journal of Health Promotion.

[bib54] Thrane C. (2006). Explaining educational-related inequalities in health: Mediation and moderator models. Social Science & Medicine.

[bib55] Toch M., Bambra C., Lunau T., van der Wel K.A., Witvliet M.I., Dragano N. (2014). All part of the job? The contribution of the psychosocial and physical work environment to health inequalities in Europe and the European health divide. International Journal of Health Services.

[bib56] Van der Lippe T., Lippenyi Z., Lössbroek J., Van Breeschoten L., Van Gerwen N., Martens T. (2016). Sustainable workforce survey.

[bib57] Van der Put A.C., Mandemakers J.J., van der Lippe T., Lippényi Z. (2019). Worksite health promotion in European organizations: Availability according to employers and employees. Investments in a sustainable workforce in Europe.

[bib58] Verra S.E., Benzerga A., Jiao B., Ruggeri K. (2019). Health promotion at work: A comparison of policy and practice across Europe. Safety and Health at Work.

[bib59] Vonneilich N., Lüdecke D., von dem Knesebeck O. (2019). Educational inequalities in self-rated health and social relationships – analyses based on the European Social Survey 2002-2016. Social Science & Medicine.

[bib60] Walters D., Wadsworth E., Quinlan M. (2013). Analysis of the determinants of workplace occupational safety and health practice in a selection of EU Member States.

