# Supplementary material for: Worksite health promotion and social inequalities in health
Source: SSM Popul Health. 2020 Jan 17;10:100543. doi: 10.1016/j.ssmph.2020.100543 (PMC6994707; doi:10.1016/j.ssmph.2020.100543)
Supplement: Supplementary material [file mmc1.docx]

**Supplementary material: results for additional analyses**

1. *Occupational status instead of education*

Occupational status was included by using the ISCO code based on asking employees to provide a full description of their occupation. These codes were transformed into occupational status scores using the ISEI transformation scale (Ganzeboom, De Graaf, & Treiman., 1992).

**Table S.1** Results for multilevel regression on self-rated health. Standard errors in parentheses.

|  | Healthy menus | | Sports facilities | | Health checks | |
| --- | --- | --- | --- | --- | --- | --- |
| Occupational status | 0.003*** | (0.000) | 0.003*** | (0.000) | 0.003*** | (0.000) |
| Healthy menus use | 0.074*** | (0.019) |  |  |  |  |
| Sports facilities use |  |  | 0.158*** | (0.024) |  |  |
| Health checks use |  |  |  |  | 0.083*** | (0.019) |
| Age | -0.176*** | (0.051) | -0.180*** | (0.051) | -0.184*** | (0.051) |
| Age*Age | 0.013* | (0.006) | 0.013* | (0.006) | 0.013* | (0.006) |
| Female | -0.059*** | (0.016) | -0.060*** | (0.016) | -0.057*** | (0.016) |
| Healthy menus availability | -0.026 | (0.022) |  |  |  |  |
| Sports facilities availability |  |  | -0.026 | (0.023) |  |  |
| Health checks availability |  |  |  |  | -0.070** | (0.024) |
| Country (ref. = NL) |  |  |  |  |  |  |
| UK | 0.087* | (0.043) | 0.071 | (0.042) | 0.072 | (0.042) |
| Germany | -0.095* | (0.041) | -0.105** | (0.040) | -0.106** | (0.039) |
| Finland | 0.094* | (0.042) | 0.034 | (0.044) | 0.105* | (0.043) |
| Sweden | 0.036 | (0.038) | -0.036 | (0.040) | 0.025 | (0.036) |
| Portugal | -0.048 | (0.038) | -0.065 | (0.037) | -0.069 | (0.038) |
| Spain | -0.097* | (0.044) | -0.121** | (0.044) | -0.125** | (0.045) |
| Hungary | -0.165*** | (0.038) | -0.176*** | (0.037) | -0.184*** | (0.038) |
| Bulgaria | 0.105** | (0.036) | 0.085* | (0.034) | 0.091** | (0.035) |
| Constant | 4.256 |  | 4.283 |  | 4.312 |  |
| Variance organisation level | 0.009 |  | 0.008 |  | 0.008 |  |
| Variance employee level | 0.513 |  | 0.512 |  | 0.513 |  |
| N employees | 249 |  | 250 |  | 251 |  |
| N organisations | 9386 |  | 9422 |  | 9458 |  |
| BIC | 29322.78 |  | 26324.72 |  | 29065.99 |  |

*p<.05, **p<.01, ***p<.001.

**Table S.2** Results for multilevel logistic regression on WHP use. Standard errors in parentheses.

|  | Healthy menus | | Sports facilities | | Health checks | |
| --- | --- | --- | --- | --- | --- | --- |
| Occupational status | 0.005* | (0.002) | 0.005* | (0.002) | -0.004* | (0.002) |
| Age | -0.336 | (0.196) | 0.088 | (0.260) | 0.425* | (0.208) |
| Age*Age | 0.024 | (0.023) | -0.025 | (0.030) | -0.023 | (0.024) |
| Female | 0.248*** | (0.063) | 0.148 | (0.081) | -0.024 | (0.065) |
| Healthy menus availability | 1.841*** | (0.196) |  |  |  |  |
| Sport facilities availability |  |  | 2.367*** | (0.236) |  |  |
| Health checks availability |  |  |  |  | 2.040*** | (0.247) |
| Country (ref. = NL) |  |  |  |  |  |  |
| UK | -1.273** | (0.402) | 0.232 | (0.451) | 0.331 | (0.475) |
| Germany | -0.226 | (0.352) | 0.888* | (0.384) | 1.478*** | (0.409) |
| Finland | 1.335*** | (0.358) | 3.331*** | (0.366) | 3.030*** | (0.419) |
| Sweden | -0.371 | (0.329) | 3.000*** | (0.332) | 1.210** | (0.381) |
| Portugal | -0.632 | (0.337) | 1.127** | (0.378) | 3.017*** | (0.383) |
| Spain | -2.217*** | (0.432) | -0.035 | (0.531) | 3.342*** | (0.432) |
| Hungary | -0.503 | (0.365) | 0.811* | (0.382) | 3.330*** | (0.408) |
| Bulgaria | -1.352*** | (0.327) | 0.501 | (0.361) | 2.469*** | (0.361) |
| N organisations | 249 |  | 250 |  | 251 |  |
| N employees | 9386 |  | 9422 |  | 9458 |  |

*p<.05, **p<.01, ***p<.001.

**Table S.3** Results for mediation analysis. Standard errors in parentheses.

|  | Direct effect | | Indirect effect | | Total effect | |
| --- | --- | --- | --- | --- | --- | --- |
| Healthy menus | 0.003*** | (0.000) | 0.000* | (0.000) | 0.003*** | (0.000) |
| Sports facilities | 0.003*** | (0.000) | 0.001* | (0.000) | 0.004*** | (0.001) |
| Health checks | 0.003*** | (0.000) | -0.000* | (0.000) | 0.003*** | (0.000) |

*p<.05, **p<.01, ***p<.001.

1. *Income instead of education*

In order to measure income, employees were asked to report their net monthly earnings from their main job in their organisation in their national currency. If respondents did not answer this question, they were asked to provide an approximation based on 21 categories specific to each country. For each category, we took the mean. All currencies were converted to euros and adjusted for purchasing power per country. Finally, we converted these monthly incomes to the natural log.

**Table S.4** Results for multilevel regression on self-rated health. Standard errors in parentheses.

|  | Healthy menus | | Sports facilities | | Health checks | |
| --- | --- | --- | --- | --- | --- | --- |
| Income (log) | 0.064*** | (0.013) | 0.062*** | (0.013) | 0.063*** | (0.013) |
| Healthy menus use | 0.083*** | (0.019) |  |  |  |  |
| Sports facilities use |  |  | 0.154*** | (0.024) |  |  |
| Health checks use |  |  |  |  | 0.070*** | (0.019) |
| Age | -0.209*** | (0.051) | -0.213*** | (0.051) | -0.215*** | (0.051) |
| Age*Age | 0.015** | (0.006) | 0.016** | (0.006) | 0.016** | (0.006) |
| Female | -0.049** | (0.016) | -0.049** | (0.016) | -0.046** | (0.016) |
| Healthy menus availability | -0.016 | (0.022) |  |  |  |  |
| Sports facilities availability |  |  | -0.016 | (0.023) |  |  |
| Health checks availability |  |  |  |  | -0.070** | (0.024) |
| Country (ref. = NL) |  |  |  |  |  |  |
| UK | 0.100* | (0.044) | 0.080 | (0.043) | 0.081 | (0.043) |
| Germany | -0.078 | (0.040) | -0.092* | (0.039) | -0.090* | (0.039) |
| Finland | 0.112** | (0.042) | 0.055 | (0.044) | 0.137** | (0.044) |
| Sweden | 0.049 | (0.038) | -0.031 | (0.040) | 0.035 | (0.037) |
| Portugal | -0.012 | (0.037) | -0.031 | (0.037) | -0.030 | (0.038) |
| Spain | -0.070 | (0.044) | -0.093* | (0.044) | -0.092* | (0.045) |
| Hungary | -0.105** | (0.039) | -0.122** | (0.039) | -0.122** | (0.040) |
| Bulgaria | 0.157*** | (0.037) | 0.133*** | (0.036) | 0.143*** | (0.037) |
| Constant | 4.002 |  | 4.049 |  | 4.078 |  |
| Variance organisation level | 0.009 |  | 0.009 |  | 0.009 |  |
| Variance employee level | 0.515 |  | 0.514 |  | 0.515 |  |
| N employees | 249 |  | 250 |  | 251 |  |
| N organisations | 9514 |  | 9549 |  | 9578 |  |
| BIC | 29728.48 |  | 26753.88 |  | 29558.87 |  |

*p<.05, **p<.01, ***p<.001.

**Table S.5** Results for multilevel logistic regression on WHP use. Standard errors in parentheses.

|  | Healthy menus | | Sports facilities | | Health checks | |
| --- | --- | --- | --- | --- | --- | --- |
| Income (log) | 0.029 | (0.027) | 0.077 | (0.059) | 0.081** | (0.055) |
| Age | -0.356 | (0.195) | 0.136 | (0.258) | 0.403 | (0.207) |
| Age*Age | 0.027 | (0.023) | -0.031 | (0.030) | -0.021 | (0.024) |
| Female | 0.275*** | (0.063) | 0.124 | (0.080) | -0.007 | (0.065) |
| Healthy menus availability | 1.893*** | (0.198) |  |  |  |  |
| Sport facilities availability |  |  | 2.395*** | (0.234) |  |  |
| Health checks availability |  |  |  |  | 2.093*** | (0.244) |
| Country (ref. = NL) |  |  |  |  |  |  |
| UK | -1.265** | (0.408) | 0.205 | (0.458) | 0.277 | (0.471) |
| Germany | -0.162 | (0.355) | 0.934* | (0.382) | 1.430** | (0.402) |
| Finland | 1.344*** | (0.361) | 3.353*** | (0.367) | 2.935*** | (0.413) |
| Sweden | -0.372 | (0.333) | 2.952*** | (0.333) | 1.178** | (0.375) |
| Portugal | -0.648 | (0.339) | 1.191** | (0.376) | 2.980*** | (0.375) |
| Spain | -2.106*** | (0.431) | -0.050 | (0.532) | 3.213*** | (0.424) |
| Hungary | -0.490 | (0.372) | 0.867* | (0.385) | 3.304*** | (0.403) |
| Bulgaria | -1.245*** | (0.331) | 0.636 | (0.361) | 2.425*** | (0.358) |
| N organisations | 249 |  | 250 |  | 251 |  |
| N employees | 9514 |  | 9549 |  | 9578 |  |

*p<.05, **p<.01, ***p<.001.

**Table S.6** Results for mediation analysis. Standard errors in parentheses.

|  | Direct effect | | Indirect effect | | Total effect | |
| --- | --- | --- | --- | --- | --- | --- |
| Healthy menus | 0.064*** | (0.013) | 0.002 | (0.005) | 0.067*** | (0.014) |
| Sports facilities | 0.062*** | (0.013) | 0.012 | (0.009) | 0.073*** | (0.016) |
| Health checks | 0.063*** | (0.013) | 0.006 | (0.004) | 0.069*** | (0.014) |

*p<.05, **p<.01, ***p<.001.

1. *Additional organisational characteristics*

Our measures for flexible working arrangements was based on a sum score of HR manager reports on whether their organisation offered employees the possibility to work at home, to work during commute and flexible starting and finishing times. The organisation’s financial situation was measured by the HR manager’s appraisal of the financial situation of the establishment over the previous three years. Competitive work culture was measured by the HR manager’s appraisal of the extent to which the establishment was results-oriented and focussed on getting the job done. Size and sector were included as sets of dummies.

**Table S.7** Results for multilevel regression on self-rated health. Standard errors in parentheses.

|  | Healthy menus | | Sports facilities | | Health checks | |
| --- | --- | --- | --- | --- | --- | --- |
| Education | 0.028*** | (0.003) | 0.028*** | (0.003) | 0.029*** | (0.003) |
| Healthy menus use | 0.078*** | (0.018) |  |  |  |  |
| Sports facilities use |  |  | 0.156*** | (0.023) |  |  |
| Health checks use |  |  |  |  | 0.084*** | (0.018) |
| Age | -0.181*** | (0.049) | -0.185*** | (0.049) | -0.183*** | (0.049) |
| Age*Age | 0.013* | (0.006) | 0.014* | (0.006) | 0.013* | (0.006) |
| Female | -0.067*** | (0.016) | -0.067*** | (0.016) | -0.064*** | (0.016) |
| Healthy menus availability | -0.028 | (0.022) |  |  |  |  |
| Sports facilities availability |  |  | -0.030 | (0.023) |  |  |
| Health checks availability |  |  |  |  | -0.060** | (0.023) |
| Flexibility arrangements | 0.010 | (0.012) | 0.008 | (0.012) | 0.009 | (0.012) |
| Financial situation | 0.025** | (0.009) | 0.026** | (0.009) | 0.026** | (0.009) |
| Competitive work culture | -0.002 | (0.011) | 0.001 | (0.011) | -0.001 | (0.011) |
| Size (ref. = large) |  |  |  |  |  |  |
| Small | -0.006 | (0.024) | -0.007 | (0.024) | -0.004 | (0.023) |
| Medium | -0.016 | (0.024) | -0.013 | (0.023) | -0.015 | (0.023) |
| Sector (ref. = health care) |  |  |  |  |  |  |
| Manufacturing | -0.007 | (0.031) | -0.012 | (0.031) | -0.007 | (0.031) |
| Higher education | -0.041 | (0.034) | -0.046 | (0.034) | -0.040 | (0.034) |
| Transport | 0.045 | (0.035) | 0.036 | (0.035) | 0.035 | (0.035) |
| Banking | 0.017 | (0.035) | 0.008 | (0.034) | 0.021 | (0.034) |
| Telecom | -0.039 | (0.039) | -0.042 | (0.038) | -0.031 | (0.038) |
| Country (ref. = NL) |  |  |  |  |  |  |
| UK | 0.095* | (0.042) | 0.081* | (0.041) | 0.081* | (0.041) |
| Germany | -0.083* | (0.039) | -0.092* | (0.038) | -0.091* | (0.038) |
| Finland | 0.099* | (0.041) | 0.043 | (0.042) | 0.105* | (0.042) |
| Sweden | 0.038 | (0.036) | -0.031 | (0.038) | 0.026 | (0.035) |
| Portugal | -0.050 | (0.037) | -0.069 | (0.036) | -0.076* | (0.037) |
| Spain | -0.043 | (0.042) | -0.069 | (0.042) | -0.076 | (0.043) |
| Hungary | -0.107** | (0.037) | -0.121** | (0.036) | -0.131*** | (0.037) |
| Bulgaria | 0.091* | (0.036) | 0.068 | (0.035) | 0.067 | (0.036) |
| Constant | 3.949 |  | 3.972 |  | 3.984 |  |
| Variance organisation level | 0.007 |  | 0.006 |  | 0.006 |  |
| Variance employee level | 0.512 |  | 0.510 |  | 0.511 |  |
| N employees | 247 |  | 248 |  | 248 |  |
| N organisations | 9885 |  | 9921 |  | 9921 |  |
| BIC | 30882.31 |  | 27861.20 |  | 30681.96 |  |

*p<.05, **p<.01, ***p<.001.

**Table S.8** Results for multilevel logistic regression on WHP use. Standard errors in parentheses.

|  | Healthy menus | | Sports facilities | | Health checks | |
| --- | --- | --- | --- | --- | --- | --- |
| Years of education | 0.025* | (0.011) | 0.027 | (0.015) | -0.024* | (0.011) |
| Age | -0.332 | (0.191) | 0.162 | (0.253) | 0.410* | (0.201) |
| Age*Age | 0.025 | (0.022) | -0.033 | (0.029) | -0.022 | (0.023) |
| Female | 0.265*** | (0.062) | 0.113 | (0.079) | 0.003 | (0.063) |
| Healthy menus availability | 1.866*** | (0.201) |  |  |  |  |
| Sport facilities availability |  |  | 2.243*** | (0.239) |  |  |
| Health checks availability |  |  |  |  | 2.055*** | (0.241) |
| Flexibility arrangements | 0.013 | (0.114) | 0.193 | (0.114) | -0.007 | (0.119) |
| Financial situation | 0.122 | (0.090) | -0.020 | (0.094) | -0.154 | (0.093) |
| Competitive work culture | 0.060 | (0.108) | -0.165 | (0.108) | -0.025 | (0.113) |
| Size (ref. = large) |  |  |  |  |  |  |
| Small | -0.584** | (0.220) | -0.302 | (0.231) | -0.493* | (0.230) |
| Medium | -0.469* | (0.233) | -0.235 | (0.227) | -0.280 | (0.240) |
| Sector (ref. = health care) |  |  |  |  |  |  |
| Manufacturing | -0.278 | (0.296) | 0.209 | (0.317) | 0.576 | (0.310) |
| Higher education | -0.469 | (0.324) | 0.305 | (0.330) | -0.016 | (0.340) |
| Transport | -0.208 | (0.333) | 0.505 | (0.354) | 0.945** | (0.349) |
| Banking | -0.198 | (0.336) | 0.455 | (0.345) | 0.161 | (0.351) |
| Telecom | 0.453 | (0.354) | 0.677 | (0.356) | 0.306 | (0.380) |
| Country (ref. = NL) |  |  |  |  |  |  |
| UK | -1.455*** | (0.413) | 0.053 | (0.449) | 0.278 | (0.472) |
| Germany | -0.132 | (0.361) | 0.991** | (0.376) | 1.464*** | (0.409) |
| Finland | 1.401*** | (0.364) | 3.210*** | (0.361) | 3.118*** | (0.415) |
| Sweden | -0.510 | (0.332) | 2.942*** | (0.326) | 1.179** | (0.372) |
| Portugal | -0.798* | (0.346) | 1.105** | (0.379) | 3.039*** | (0.380) |
| Spain | -2.164*** | (0.432) | 0.005 | (0.512) | 3.242*** | (0.424) |
| Hungary | -0.592 | (0.372) | 0.976** | (0.376) | 3.272*** | (0.400) |
| Bulgaria | -1.356*** | (0.343) | 0.739* | (0.368) | 2.651*** | (0.376) |
| N organisations | 247 |  | 248 |  | 248 |  |
| N employees | 9885 |  | 9921 |  | 9921 |  |

*p<.05, **p<.01, ***p<.001.

**Table S.9** Results for mediation analysis. Standard errors in parentheses.

|  | Direct effect | | Indirect effect | | Total effect | |
| --- | --- | --- | --- | --- | --- | --- |
| Healthy menus | 0.028*** | (0.003) | 0.002* | (0.001) | 0.030*** | (0.003) |
| Sports facilities | 0.028*** | (0.003) | 0.004 | (0.002) | 0.033*** | (0.004) |
| Health checks | 0.029*** | (0.003) | -0.002* | (0.001) | 0.027*** | (0.003) |

*p<.05, **p<.01, ***p<.001.

1. *Number of WHP*

We measured the number of WHP employees used by creating a sum score of their use of healthy menus, sports facilities and health checks.

**Table S.10** Results for multilevel regression on self-rated health. Standard errors in parentheses.

|  | Health | |
| --- | --- | --- |
| Income | 0.027*** | (0.003) |
| Number of WHP used | 0.085*** | (0.010) |
| Age | -0.189*** | (0.049) |
| Age*Age | 0.014* | (0.006) |
| Female | -0.065*** | (0.015) |
| Healthy menus availability | -0.026 | (0.021) |
| Sports facilities availability | -0.025 | (0.023) |
| Health checks availability | -0.065** | (0.023) |
| Country (ref. = NL) |  |  |
| UK | 0.084* | (0.041) |
| Germany | -0.117** | (0.037) |
| Finland | 0.033* | (0.043) |
| Sweden | -0.004 | (0.038) |
| Portugal | -0.089* | (0.036) |
| Spain | -0.074 | (0.043) |
| Hungary | -0.146*** | (0.037) |
| Bulgaria | 0.066 | (0.034) |
| Constant | 4.103 |  |
| Variance organisation level | 0.007 |  |
| Variance employee level | 0.510 |  |
| N employees | 249 |  |
| N organisations | 9991 |  |
| BIC | 32662.74 |  |

*p<.05, **p<.01, ***p<.001.

**Table S.11** Results for multilevel logistic regression on WHP use. Standard errors in parentheses.

|  | Nr of WHP used | |
| --- | --- | --- |
| Years of education | -0.012 | (0.010) |
| Age | -0.009 | (0.171) |
| Age*Age | -0.002 | (0.020) |
| Female | 0.112* | (0.055) |
| Healthy menus availability | 0.696*** | (0.185) |
| Sport facilities availability | 0.875*** | (0.205) |
| Health checks availability | 0.728*** | (0.199) |
| Country (ref. = NL) |  |  |
| UK | -1.025** | (0.366) |
| Germany | -0.384 | (0.321) |
| Finland | 2.825*** | (0.396) |
| Sweden | 1.164*** | (0.323) |
| Portugal | 1.352*** | (0.315) |
| Spain | 1.570*** | (0.372) |
| Hungary | 1.076** | (0.340) |
| Bulgaria | 0.612* | (0.298) |
| N organisations | 249 |  |
| N employees | 9991 |  |

*p<.05, **p<.01, ***p<.001.

**Table S.12** Results for mediation analysis using occupational status. Standard errors are shown in parentheses.

|  | Direct effect | | Indirect effect | | Total effect | |
| --- | --- | --- | --- | --- | --- | --- |
| Number of WHP used | 0.027*** | (0.003) | -0.001 | (0.001) | 0.026*** | (0.003) |

*p<.05, **p<.01, ***p<.001.

**References**

Ganzeboom, H.B.G., De Graaf, P.M., Treiman, D.J., 1992. A standard international socio-economic

index of occupational status. *Social Science Research, 21*, 1–56. https://doi.org/10.1016/0049-089X(92)90017-B.
